# Sulphate of Bebeerine in Diarrhœa

**Published:** 1855-02

**Authors:** 


Sulphate of Bebeerine in Diarrhœa.—Mr. Mathews, of Manchester, strongly recommends in the *Lancet* the use of sulphate of bebeerine as a remedy for severe diarrhœa, in fact, he states that he has found it "a very near approach to a specific, and that with a very few doses, as a rule; indeed, in many instances, its effects are magical, for in the course of half an hour I have seen a patient restored from intolerable anguish to perfect ease." He generally first gives a pill, consisting of two grains of calomel and half a grain of opium, and if there is great pain, or the vomiting is urgent, orders a sinapism to the scrobiculus cordis, and then follows up with the following mixture:—

Sulphate of bebeerine, twelve grains; sulphuric acid and rectified ether, of each, twelve minims; cinnamon water, six ounces; mix. One ounce to be taken every four hours.—*Dublin Hospital Gazette.*

